# Identifying associations between pig pathologies using a multi-dimensional machine learning methodology

**DOI:** 10.1186/1746-6148-8-151

**Published:** 2012-08-31

**Authors:** Manuel J Sanchez-Vazquez, Mirjam Nielen, Sandra A Edwards, George J Gunn, Fraser I Lewis

**Affiliations:** 1 Scottish Agricultural College, Kings Buildings, West Mains Road, Edinburgh, EH9 3JG, UK; 2 Department of Farm Animal Health, Faculty of Veterinary Medicine, Utrecht University, Utrecht, The Netherlands; 3 Newcastle University, Agriculture Building, Newcastle upon Tyne, NE1 7RU, Newcastle, UK; 4 Section of Epidemiology, Vetsuisse Faculty, University of Zurich, Zurich, Switzerland

## Abstract

**Background:**

Abattoir detected pathologies are of crucial importance to both pig production and food safety. Usually, more than one pathology coexist in a pig herd although it often remains unknown how these different pathologies interrelate to each other. Identification of the associations between different pathologies may facilitate an improved understanding of their underlying biological linkage, and support the veterinarians in encouraging control strategies aimed at reducing the prevalence of not just one, but two or more conditions simultaneously.

**Results:**

Multi-dimensional machine learning methodology was used to identify associations between ten typical pathologies in 6485 batches of slaughtered finishing pigs, assisting the comprehension of their biological association. Pathologies potentially associated with septicaemia (e.g. pericarditis, peritonitis) appear interrelated, suggesting on-going bacterial challenges by pathogens such as *Haemophilus parasuis* and *Streptococcus suis*. Furthermore, hepatic scarring appears interrelated with both milk spot livers (*Ascaris suum*) and bacteria-related pathologies, suggesting a potential multi-pathogen nature for this pathology.

**Conclusions:**

The application of novel multi-dimensional machine learning methodology provided new insights into how typical pig pathologies are potentially interrelated at batch level. The methodology presented is a powerful exploratory tool to generate hypotheses, applicable to a wide range of studies in veterinary research.

## Background

Abattoir post-mortem inspection offers good opportunities for pig health monitoring [1] and it has been widely used as a data source for epidemiology-based analyses. Most of these studies focus on the identification of risk factors influencing the presence of the major abattoir pathologies: pneumonia, pleurisy and milk spot liver [2-9]. Few reports investigate how the different pathologies are interrelated [1,10,11]. Identification of the associations between pathologies may assist in elucidating theories on their biological connection and could greatly contribute to facilitating their control – for example by encouraging veterinarians to establish intervention strategies aimed at reducing the prevalence of not just one, but two or more conditions simultaneously. Knowledge of associations between lesions could also be employed to inform official abattoir inspection systems, in which the presence of one pathol ogy could trigger an inspection for others.

Official routine meat inspections are implemented world-wide with the main objective of ensuring food safety. This system, however, is imperfect and is particularly lacking in sensitivity [12,13]. Pig health schemes were proposed to provide an integrated system to capture abattoir information based on more detailed post-mortem inspection [14] which is considered to improve classification characteristics, particularly sensitivity [12]. Good examples of these initiatives in Europe are the British pig health schemes. On a regular basis, swine specialists carry out detailed post-mortem examinations in parallel to the official food-safety routine meat inspections. These schemes monitor the presence of various pathologies detected by means of a detailed inspection of the pluck and the skin of the slaughtered pig. These pathologies are normally associated with a reduction in performance traits or are potential indicators of the presence of welfare problems in the herds [10,15-17].

Graphical modelling has been increasingly used in veterinary epidemiology to investigate and express the relationships between factors influencing diseased/unproductive status in livestock [18-23]. Frequently, studies utilising graphical models are based on structure discovery approaches, which are data-driven multivariate methodologies resulting in graphical outputs such as networks or path/chain models. Structure discovery has been employed to explore how mastitis and fertility management influence production in dairy herds [18]; to identify changes in pig behaviour related to early piglets mortality [19]; to investigate the most likely pathogens involved in clinical mastitis in dairy cows [20]; and to identify those farm risk factors associated with bovine viral diarrhoea [23]. Besides these examples, other studies employed graphical models informed using existing/expert knowledge to describe risk factors influencing the prevalence of Mycoplasma hyopneumoniae [21]; and to estimate the risk of leg disorders in finishing pigs [22]. A crucial distinction among the abovementioned papers, is that these two latter studies [21,22] did not use structure discovery to inform structure of the network, but were rather based on published knowledge and expert opinion. The latter is highly subjective and if, as in this study, extensive data are available, then extracting the co-dependence network structure from observed data provides objective and robust empirical analyses.

Multi-dimensional machine learning methodology (also known as Bayesian graphical modelling) is a variety of graphical modelling structure discovery techniques used to identify the dependency structure that encodes the joint probability distribution between variables [24,25], allowing for both visualization and estimation of associations. In short, this process consists of a series of model searches to identify the multi-dimensional model that best explains the data, using Bayes factors to compare between models [23]. This approach allows estimation of the associations between variables and distinguishes between direct and indirect dependence [25] (dependence being equivalent to biological association), contributing to generate hypotheses about the nature of the interrelationships. Multi-dimensional machine learning methodology offers an intuitively appealing and technically elegant way to investigate multiple associations between variables compared to more conventional multivariate statistical approaches (e.g. principal component and factor analyses). This methodology is used extensively in fields such as bioinformatics and genetics [26-28] and only recently has been applied in the veterinary field [23].

This paper uses a multi-dimensional machine learning methodology to identify whether associations exist between the different pathologies reported by the British pig health schemes. The results of this study could assist veterinarians in the control of these conditions by implementing strategies to control several conditions at once. These results could be also utilised to review current pig abattoir inspection strategies, and inform more targeted risk based inspections. Farmed pigs are normally considered as a grouped unit, where complex interactions take place between the environment, mainly determined by the housing system and the husbandry practices, and the pigs, characterised by their genetics, idiosyncratic behaviour and baseline health status [29]. For these reasons this study focuses on the interrelationship occurring between pathologies at batch level.

## Methods

### Data source

Abattoir data were accessed through the databases of the two pig abattoir lesion scoring health schemes which exist in Great Britain: Wholesome Pigs Scotland (WPS) (covering Scotland) and British Pig Health Scheme (BPHS) (covering England and Wales) [6]. The health schemes provide services in 17 pig abattoirs. Both schemes obtain a sample from each batch of pigs by assessing every second pig on the slaughter line. The scoring was carried out by swine veterinarians trained in this method of testing on the abattoir inspection line. The data were from a three year period (July 2005 to June 2008).

### Dataset

For the purpose of this investigation, a batch is defined as a group of pigs from a single farm submitted to the abattoir on a particular date. A total of 6485 batches were included, submitted from 1138 farms, with a median of 4 batches assessed per farm (first quartile 2, third quartile 8). All the batches consisted of exactly 50 pigs assessed.

### Scoring for the different pathologies

Ten pathologies reported by the health schemes are included in this study: Enzootic-pneumonia-like lesions, pleurisy (pleuritic lesions), milk spots, hepatic scarring, pericarditis, peritonitis, (lung) abscess, pyaemia (pyaemic lung lesions), tail damage and papular dermatitis. A further explanation on the gross pathology description, the most typical cause associated and the scoring system for each condition are presented in Table 1. In this study, a positive case for each pathology was defined as a pig affected with any degree of lesion and a negative when lesions were absent.

**Table 1:** Summary of the gross pathology description of conditions studied with their most typical cause and the scoring system

**Pathology**	**Gross pathology and most typical cause**	**Scoring system**
Enzootic pneumonia-like lesions	A red-tan-grey discoloration, collapse, and rubbery firmness affecting cranioventral regions of the lungs in a lobular pattern. *Mycoplasma hyopneumoniae* is the causal infectious agent [42].	Represent the approximate percentage of lung with consolidation. Scale from 0 to 55 in 5 steps.
Pleurisy	Fibrous/fibrinous pleural adhesions. Can be associated with *Actinobacilus pleuropneumoniae*, *Pasteurella* spp*, Mycoplasma hyorhinis*, *Mycoplasma hyopneumoniae,* swine influenza and *Haemophilus parasuis*[4]. Focal areas of bronchopneumonia with overlying pleurisy often associated with *A. pleuropneumoniae* usually affecting the middle or caudal lung lobes [42].	Three categories represent severity of the lesion with baseline absence. Binary, present or absent.
Milk Spots	Whitish foci, occurring in the liver stroma when *Ascaris suum* larvae are immobilised by the host’s inflammatory reaction [39].	Binary, present or absent.
Hepatic scarring	Mild fibrotic lesions affecting the capsule of Glisson, with no liver parenchyma alteration. Possibly associated with healed *Ascaris suum* lesions.	Binary, present or absent.
Pericarditis	Inflammation of the pericardium, usually fibrinous. Unspecific condition that could be associated with bacterial diseases, e.g. Glasser’s disease and pasteurellosis [43].	Binary, present or absent.
Peritonitis	Fibrous/fibrinopurulent lesions typically associated with *Arcanobacterium pyogenes* and *Escherichia coli.* Serofibrinous lesions associated with *Haemophilus parasuis* (Glasser’s disease) and *Streptococcus suis*[34,35].	Binary, present or absent.
Abscess	Localised/encapsulated collection of pus within the lung. Various pathogens involved, typically *Arcanobacterium pyogenes*[10].	Binary, present or absent.
Pyaemia	Multiple small abscesses in the lung parenchyma. Pyaemic spread of infection from other focus: *Arcanobacterium pyogenes* frequent involved [10].	Binary, present or absent.
Tail damage	Presence of old or recent tail lesions. Typically associated with tail biting [17].	Binary, present or absent.
Papular dermatitis	Reddish papules/nodules found on belly, head and buttocks or widespread across the skin, depending on the severity. This lesion is potentially associated with Sarcoptic mange [40].	Three categories: accounting for severity and distribution of the skin lesions.

### Consistency in the scoring of the pathologies

Both health schemes carried out exercises to standardise the definition of each lesion across the inspectors. One WPS assessor was involved in the training of all the other inspectors that carried out WPS and BPHS assessments during the three year period included in this study. Once a year, all the inspectors underwent a refresher/training day where the same pigs and pathologies were assessed by all the assessors and feed-back was provided by the trainer. These assessment exercises aimed to maintain the consistency in the scoring criteria across assessors by identifying and correcting potential misclassifications. Furthermore, the schemes aimed to include at least two assessors per abattoir and to place each assessor in at least two different abattoirs, thereby minimising the potential of operator bias.

### Definition of pathology batch-status variables

The machine learning approach utilised requires working with categorical variables. Batches were categorised into lesion present/absent using the frequency distribution of the batch prevalence for the different pathologies to determine data-derived cut-off points (further details are provided in Additional file 1: Figure S1 and Figure S2). In the context of this study, where all the batches have the same number of pigs inspected (i.e. 50), frequency and proportion are equivalent and the cut–offs are defined in terms of frequencies per batch. For enzootic pneumonia-like lesions, three categories were identified based on within batch prevalence: EP high) when more than 25 pigs were affected with any degree of severity; EP moderate-low) when between one and 25 pigs were affected; and EP zero) when no pigs were affected. For pleurisy, thee categories were also identified based on within batch prevalence: PL high) when more than seven pigs were affected; PL moderate-low) when between one and seven pigs were affected and PL zero) when no pigs were affected. The three prevalence level categories identified for enzootic pneumonia-like lesions and pleurisy were each separated into three binary variables (e.g. EP high [yes, no], and so on) to reflect the pathology batch-status. Splitting the prevalence level categories into three binary variables was chosen over creating a single multinomial variable to add flexibility in the modelling and facilitate the interpretation of the model outputs. For the other pathologies which have a much lower prevalence (i.e. milk spots, hepatic scarring, pericarditis, peritonitis, papular dermatitis, tail damage, abscess and pyaemia) batches were considered positive if at least one pig was found affected, and negative otherwise. In summary, the ten different pathologies were studied through 14 binary variables reflecting the pathology batch-status. A data break-down of the frequencies for the pathology batch-status variables is presented by pairs in Table 2.

**Table 2:** The break-down of the frequencies of the variables expressing batch-status for the different pathologies studies by pairs, N = 6485 batches of slaughtered pigs

		**EP**			**PL**			**MS**	**HS**	**PC**	**PT**	**Abs.**	**Pya.**	**Tail**
		**high**	**M/L**	**zero**	**high**	**M/L**	**zero**							
	**high**	656	1124	46	-	-	-	-	-	-	-	-	-	-
**PL**	**M/L**	614	2679	437	-	-	-	-	-	-	-	-	-	-
	**zero**	80	642	207	-	-	-	-	-	-	-	-	-	-
**MS**		417	1327	196	508	1163	270	-	-	-	-	-	-	-
**HS**		842	2544	336	1038	2158	526	1328	-	-	-	-	-	-
**PC**		986	2970	305	1531	2393	337	1279	2550	-	-	-	-	-
**PT**		259	694	82	419	541	75	357	808	860	-	-	-	-
**Abscess**		326	851	55	564	598	70	387	732	882	228	-	-	-
**Pyaemia**		154	362	26	243	267	32	174	383	388	172	171	-	-
**Tail**		109	353	75	180	316	41	137	275	362	136	115	126	-
**PD**		344	1008	106	471	774	213	516	959	1009	269	319	136	144

Abbreviations/initials: EP, enzootic pneumonia-like lesions; PL, pleurisy; MS, milk spots; HS, hepatic scarring, PC, pericarditis; PT, peritonitis; Abs., abscess; Pya., pyaemia; Tail, tail damage; PD, papular dermatitis; M/L, moderate/low.

### Multi-dimensional machine learning methodology

**Figure 1:**
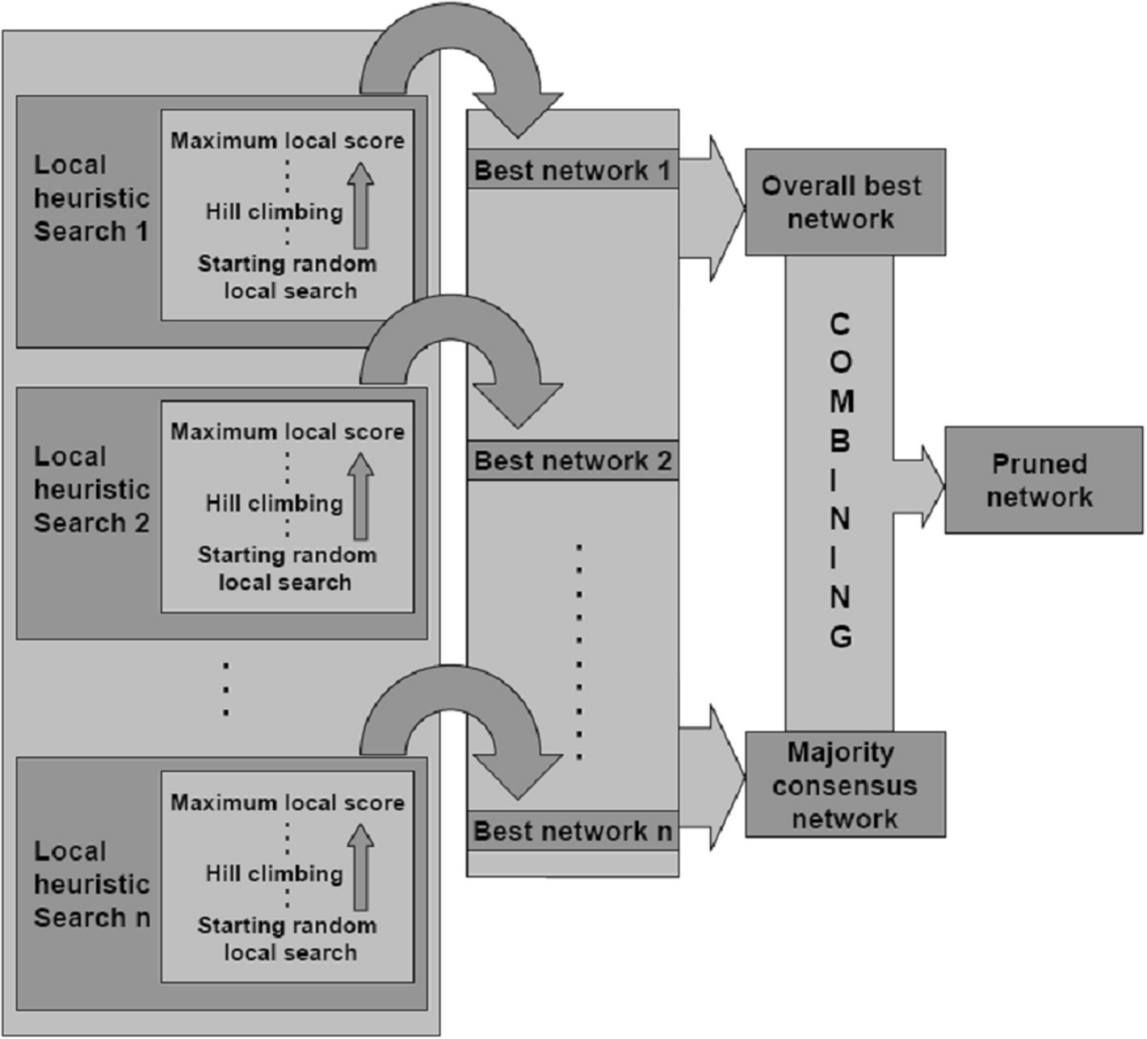
**Diagram representing the machine learning structure discovery steps.** The process starts with local searches, where local best networks are recruited, then the overall best network and the majority consensus network are identified. Finally the combination of the two latter structures leads to the pruned network.

The process explained below aims to identify an optimal multi-dimensional model, i.e. a graphical model displayed as a network of connections, where each connection (arc) describes a statistically significant association between the different lesions in the data. Figure 1 schematically represents the machine leaning structure discovery process utilised, which is initiated with numerous series of searches followed by steps to summarise the results of each search. This methodology consists of fitting models which are network structures technically referred to as directed acyclic graphs (a graph with no loops), in which nodes correspond to the pathology batch-status variables and arcs between nodes (represented by arrows) indicate that a direct probabilistic dependency (e.g. an association) exist between nodes.

### Direction of the arrows

The direction of the arcs connecting nodes is informed by the data, reflecting the dependency structure which generated the data [26]. The direction only implies association and says nothing of causality. Arc direction is as a result of the underlying mathematics used to construct the models (technically the graph denotes a factorisation of the joint probability distribution of the data). Models with particular arc directions may be better fit to the data than with the reversed directions, and therefore preferred, however, it would be incorrect with this information alone to infer that the biological dependence between two nodes is supported more in one direction over another, all that can be inferred is that association exists between nodes.

### Searching for locally optimal structures

The machine learning structure discovery process was performed through series of local heuristic searches using a standard approach proposed by Heckerman et al. [24]. Locally optimal models are identified by random-restart local hill climbing searches, also known as a “greedy search” [26], which seek to maximise the goodness of fit metric (network score) for each model. This network score is given by the (log) marginal likelihood of the data given the model; equivalent to the Bayes factor when using equal prior on each model structure. This search process can be thought of as roughly analogous to stepwise regression in linear modelling but conducted in multiple dimensions where the initial model from which the search commences is randomly chosen. The interrelationships within different batch-status categories for the same pathology are inversely related – i.e. when one batch-status is present the others are not. Therefore arcs connecting the different batch-status categories for the same pathology (e.g. EP zero with EP high, or EP high with EP mild/moderate, and so on) were banned from the search.

### Summarising the results from the local searches

Alternative and competing explanations of the data are produced during the local search process; different local searches may lead to different structural features (e.g. arcs) that appear in some networks but not in others [25]. A great deal of commonality across the search results is expected and strong features should be extracted reliably [25]. The aim is to produce an optimal structure that robustly represents the main associations. Three main ways are proposed to summarise the results from the local searches:

(1) The “overall best network” is the single structure with the best score (according to the Bayes factor) across all the searches. This structure identifies the potential pathways (composed of sets of arcs) of associations between variables. Some of these pathways may be weak, however, i.e. only identified for this particular network and may incur over-fitting; a common problem within structure discovery approaches [26].

(2) The “majority consensus network” is the structure that represents those common features present in the majority of the best-scored networks identified across all the heuristic searches. By using this, those associations (arcs) that were present in the majority (over 50%) of all the locally best networks were kept. This approach is typically employed in phylogenetic studies [30] and it has been suggested for structure discovery [23].

(3) The “pruned network” is the structure that combines the two approaches mentioned above to produce a more robust output. Only those arcs that were part of the overall best network and also recruited by the majority consensus network were kept. Lewis et al. [23] proposed this approach mimicking pruning performed in decision tree inferences, which is essential to reduce over-fitting [31].

### Identifying the final network

Out of the three structures described above, the pruned network is the model that provides the most robust and conservative approach and is therefore considered in this paper as the principal result. The strength of the association between two nodes (pathology batch-status variables) present in the pruned network was estimated by calculating the relative risk (RR) (also known as risk ratio) [32]. RR is calculated as the proportion of batches affected with condition A given condition B is present in the batch, divided by proportion of batches with the condition A but with condition B not present. The 95% confidence intervals (CI) for the RR were estimated using Monte-Carlo simulation.

### Parameters in the search algorithm

Three major characteristics define the algorithm of the heuristic search:

set.seed a single value that sets the starting point for the search.

i.permutations a number that defines the times an initial empty network is perturbed to construct a random network from which a stepwise search is performed.

max.parents a number between 2 and total number of variables minus 1. This number defines the maximum number of arcs reaching a particular node. In this study no restrictions were placed upon the number of parents and the maximum, 13, was allowed in all searches.

The optimal number of local searches required to identify a robust machine learning structure is problem specific. In this study, the number was determined empirically by running two parallel sets of searches, differing in the set.seed value. The number of local searches was increased until both sets reached the same majority consensus network, thereby suggesting that a sufficient numbers of searches had been run to provide robust outputs. The results from both sets of searches were pooled to identify the best overall single network which, combined with the majority consensus network, led to the pruned network.

The analyses were performed in R [33] using a library written by FIL (freely available upon request) to perform the structure search. Other broadly similar libraries are available for use within R from CRAN (Comprehensive R Archive Network) website, and similar toolboxes are available for use with MATLAB.

## Results

Empirical investigation determined that 10000 local searches were sufficient to ensure robust modelling results.

### Graphical outputs

**Figure 2:**
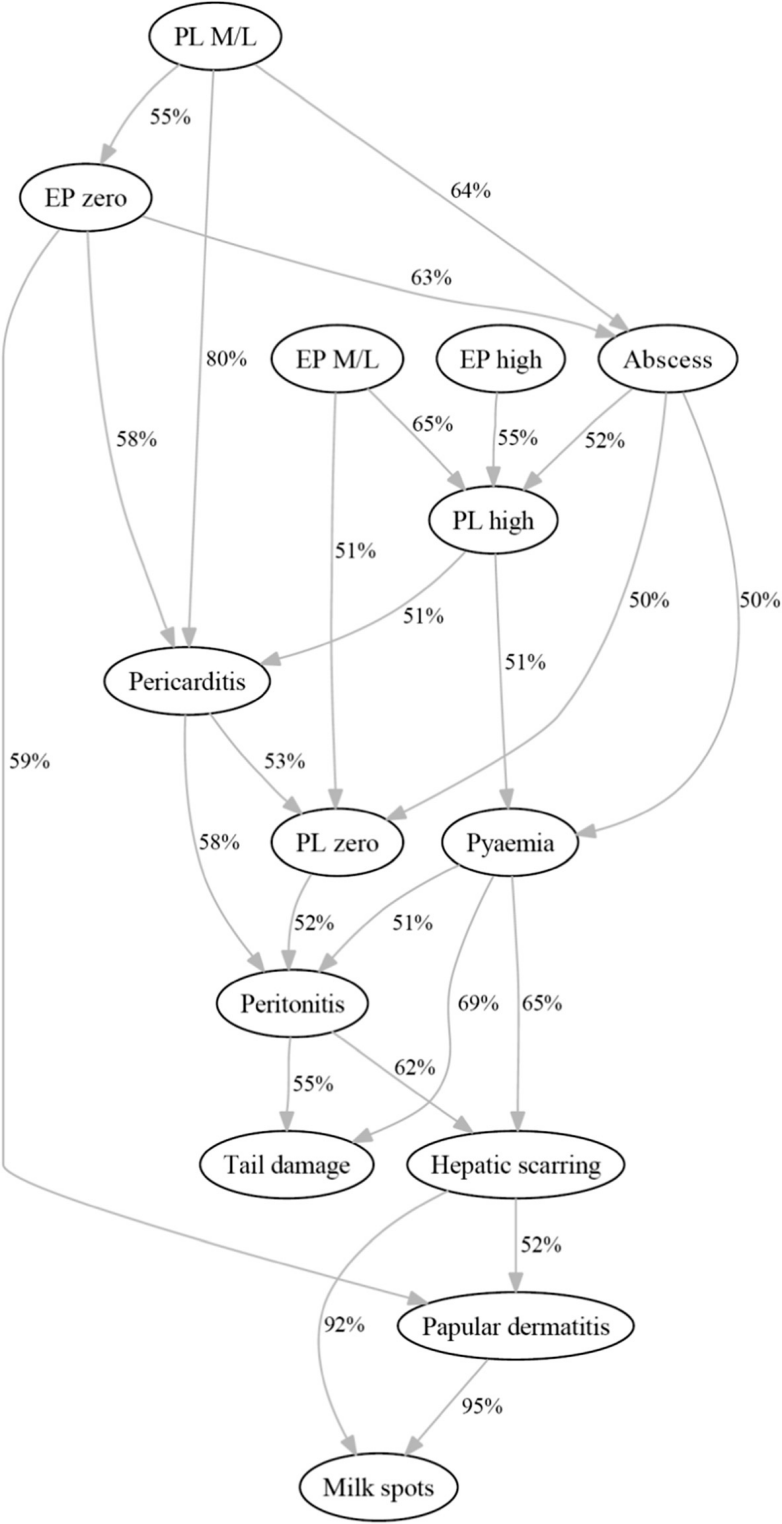
**Associations between the variables expressing the batch-status for the different pathologies as identified by the majority consensus network, N = 6485 batches of slaughtered pigs.** This network encloses the joint probability of the pathology batch-status variables with the arrows representing the associations between them (pointing in the direction reflected by the data structure). The figures beside the arrows represent the percentages of local searches in which the arc appears.

**Figure 3:**
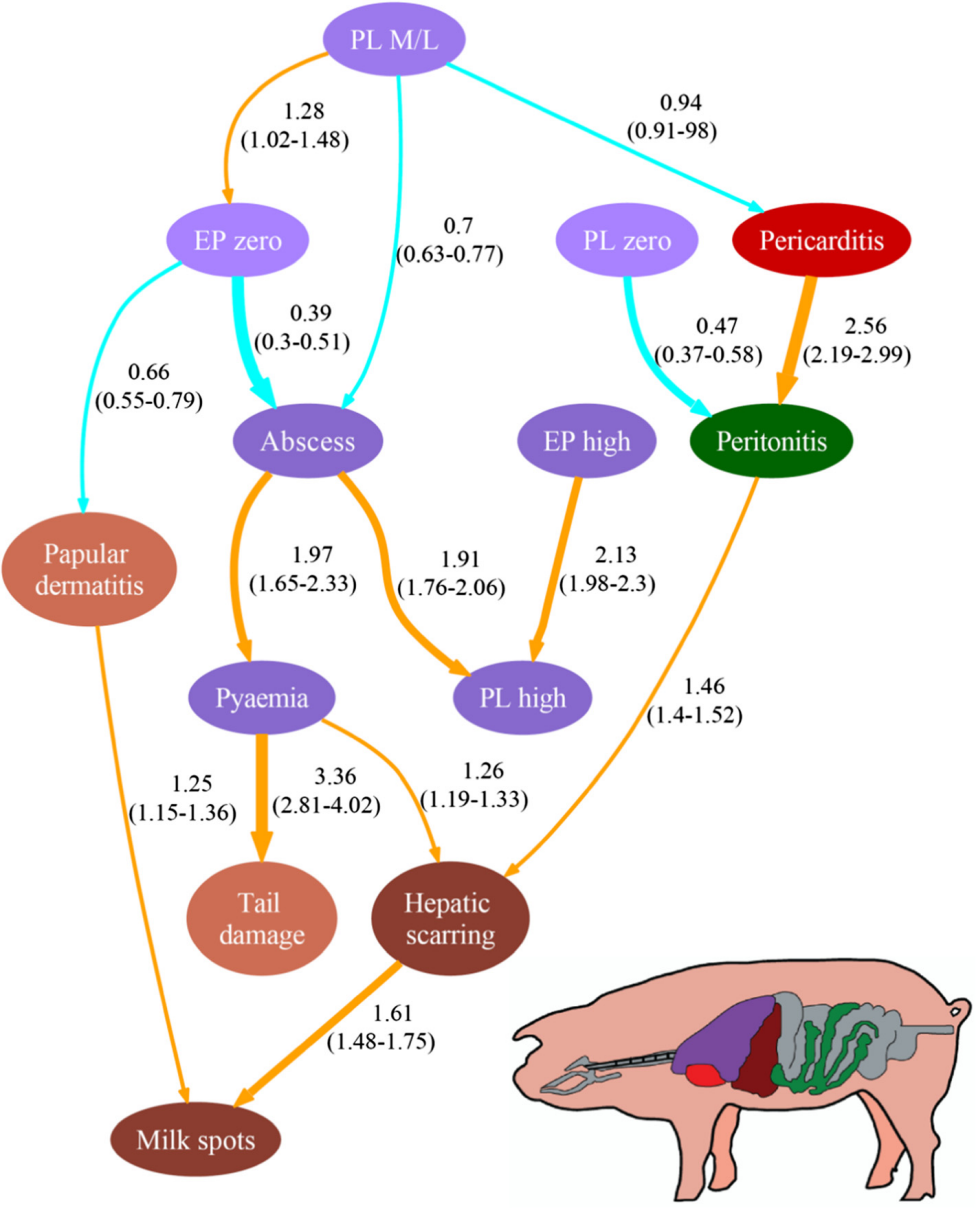
**Associations between the variables expressing the batch-status for the different pathologies as identified by the pruned network, N = 6485 batches of slaughtered pigs.** This network encloses the joint probability of the pathology batch-status variables with the arrows representing the associations between them (pointing in the direction reflected by the data structure). The figures beside the arrows represent the estimated relative risk (RR) reflecting the strength of the association (the figures between brackets represent the 95% confidence intervals). The thickness of the arrows reflects the strength of the association. The thinnest arrows represent mild associations (RRs between 0.66-1 and 1–1.5); the intermediate thickness represents moderate associations (RRs between 0.5-0.66 and 1.5-2.5); and the thickest ones represent strong associations (RRs less than 0.5 and over 2.5). The arrows in orange represent positive association (RRs > 1) and the arrows in blue represent negative associations (RRs < 1). To facilitate the visualization the variables are colour-coded according to the organs they are attributed to: purple for lungs, red for the heart, brown for the liver, green for the peritoneum, and pink for the skin/tail. Colour gradients are used for enzootic pneumonia and pleurisy batch-status variables to indicate the different levels of prevalence (high, moderate/low and zero).

The “majority consensus network” is presented in Figure 2 and provides complementary information to the main output from this investigation, the “pruned network”, which is presented in Figure 3 completed with estimated RRs. The arcs presented in the “pruned network” could be identified in the “majority consensus network” to determine the percentage of local searches in which the particular arc appears, informing about the robustness. Thus, the “majority consensus network” (Figure 2) shows that the connections leading to milk spots from hepatic scarring and papular dermatitis, are the most robust – present in more than 90% of searches. In the pruned network (Figure 3) these arcs are retained, and it is observed that those batches with hepatic scarring had a moderate risk of milk spots compare to those batches not presenting hepatic scaring; likewise batches with papular dermatitis had a milder risk of milk spots compare to those with papular dermatitis absent. Figure 3 also shows that batches with mild or moderate levels of pleurisy were more likely to be enzootic pneumonia-like free than those with other category levels of pleurisy. The pneumonia free batches were more likely to be also free of papular dermatitis than those with pneumonia. Batches with a high level of enzootic pneumonia-like lesions had a moderate risk (i.e. RRs between 1.5 and 2.5) of having a high level of pleurisy compare to those batches with pneumonia absent or with moderate/low level. Having abscesses is associated with batches with a higher level of pleurisy compare to those with no abscesses. Batches with a moderate/low level of pleurisy had a negative risk of abscess and pericarditis compare to other batches with other levels of pleurisy. There is stronger risk (i.e. RRs over 2.5) of having peritonitis if pericarditis is present in the batch than if it is absent; conversely those batches with zero level of pleurisy are more likely to be peritonitis free than those with pleurisy present. Batches with peritonitis also had a milder risk (i.e. RRs between 1 and 1.5) of hepatic scarring compare to batches without peritonitis. Batches with pyaemia had a mild risk of hepatic scarring and a strong risk of having tail damage compare to those batches with pyaemia absent.

## Discussion

This paper describes the application of a multi-dimensional machine learning methodology to multivariate epidemiological analyses. Applying this methodology to the data comprising of the typical pathologies present in slaughtered finishing pigs has led to an easy to interpret, highly visual, and statistically robust output: a network in which the main associations between the pathologies are easily identifiable.

### The interrelationship between the pathologies

This study has provided information on the nature of hepatic scarring which is thought to be a post healing stage of milk spots; but for which other aetiologies can not be discarded. The results suggest that both *Ascaris suum* and systemic bacterial infections are independently interrelated with the presence of the liver capsule scarring. The former is reflected in the moderate association with milk spots, which seems to be a highly robust interrelationship as it was recruited in 92% of the searches. Different stages of *A. suum* parasitism within the same batch may take place; leading to coexistence of active milk spot lesions with those already healed, i.e. hepatic scarring. The potential bacterial aetiology of hepatic scarring is suggested as its risk increases with the presence of pyaemia – suggesting that both pathologies may be associated with *Arcanobacterium pyogenes*. In addition, hepatic scarring appears interrelated with peritonitis, which is typically present in systemic infections by *Haemophilus parasuis* (responsible for Glasser’s disease) or *Streptococcus suis*[34,35]. These latter infectious agents would also explain the positive association between peritonitis and pericarditis. Likewise it was observed that when pleurisy was absent the chance of being peritonitis free increased.

Severe pneumonic pasteurellosis is typically manifested by abscessation and thoracic wall adherences [36] which explains the associations detected between pleurisy and abscess. Conversely, absence of pneumonia is associated with lower levels of abscesses. High batch prevalence of enzootic pneumonia-like lesions is interrelated to high levels of pleurisy, which is an expected finding as both respiratory conditions share common husbandry risk factors [6] and *Mycoplasma hyopneumoniae* (main pathogen for enzootic pneumonia) contributes to the occurrence of pleurisy [4]. This latter association may reflect the presence of poor health levels, particularly in the control of respiratory diseases. Alternatively, batches with mild or moderate levels of pleurisy appear more likely to be free of enzootic pneumonia, pericarditis and abscess, reflecting perhaps high health batches. Papular dermatitis is associated with the presence of milk spots, both being parasitic conditions. This association could reflect poor parasitic control strategies for some producers and highlights the fact that, even with current systems of production, parasitism is still neglected by some sectors of the industry. These results could be used to optimise abattoir inspection strategies. For example, when papular dermatitis is detected in the pigs (e.g. during the ante mortem inspections) the meat inspectors should place more emphasis in the liver inspections of those batches. This would be a proxy for the implementation of risk based surveillance abattoir polices that could optimise the use of industry and government resources [37].

Presence of pyaemia in the batch is associated with presence of tail damage. This latter pathology is known to be involved in early stages of the pathogenesis of pyaemia, by facilitating an entry access for bacteria [10]. At pig level these two lesions might not coexist simultaneously due to the time gap between the tail damage and the development of the pyaemia [10], but a batch level investigation may have assisted to find such association. The approach used in this study, investigating batch level prevalence, not only maintains coherence with the nature of pig production, but would have also assisted in the identification of any association when two pathologies may be part of the same causal pathway (e.g. milk spots and hepatic scarring). In this scenario it is likely that the pathologies do not coexist in the same pig, therefore pig level investigation would be an inefficient way of exploring their association. Furthermore, pathologies presented in a mild form or during the healing process can be missed in the abattoir inspections; whereas if they are present in more than one pig, the chance of being detected by the abattoir assessors increases leading to a more adequate batch level classification.

### Clustering in the structure of data

In this study the impact on the analyses of the potential clustering structure in the data has been mitigated by modelling the data at batch level. Batch is typically the lowest and likely the strongest level of clustering present in abattoir data [38], particularly in health scheme pig abattoir data [6,8]. It is also arguable that for the type of analyses presented – particularly the multi-dimensional aspect– clustering is of far less concern than in other types of traditional statistical analyses. For other potential levels of clustering to be an issue, e.g. on-farm (or abattoir or season), this would require that on different farms the proportion of batches which have, for example, {lesion A present given that lesion B is present and lesion C is not present and lesion D is present and so on…} are substantially different, and similarly for all the other conditional probabilities in the model. This form of "group-effect" is unlikely to be sizable after having already jointly adjusted for all the other conditions present in a batch. Hence, intuitively it could be argued that the machine learning methodology is robust to clustering, whether this is at farm/abattoir/season level. In practical terms, this assertion cannot be rigorously tested with this methodology and it should therefore be acknowledged as a potential limitation in this study.

### Constraints of abattoir gross pathology data

The different pathologies were presented in this paper with their most typical cause (Table 1) and although some of them, i.e. EP-like lesion, milk spots and papular dermatitis, can be considered good proxies for specific pathogens [9,15,39,40], none of them are strictly pathognomonic. The data obtained from abattoir monitoring carried out by the health schemes offered here a unique opportunity to explore the associations between these relevant pig pathologies. The presence of operator bias across the assessors, affecting the gross pathology classification cannot be absolutely ruled out, but the definition of the lesions did not change during the period included in our study. Additionally, the health schemes organise training and refresher days for the veterinarians and conduct internal comparisons on the same pigs assessed by different veterinarians, aiming to maintain assessor consistency over time

The results from this study are applicable to the whole study population, i.e. those farms participating in the pig health schemes, and particularly to those units that submitted several batches of pigs over the time period included in the study. Additionally, the results could be extrapolated to the population of British pig commercial units, as the assessments carried by the health schemes are considered representative of the British commercial sector [41].

### Further discussion on the structure discovery approach

The multi-dimensional machine learning methodology presented is well suited for investigating multiple associations between pathologies, generating hypotheses about potential interrelationships. Linear models and their generalizations, for example, would have required designating one variable as a response and modelling the rest as a set of independent predictors. Multivariate techniques like principal component and factor analyses utilise dimension reduction to facilitate the identification of uncorrelated subgroups of variables (i.e. principal components and factors). In contrast, machine learning structure discovery does not reduce the dimensions of the data and its graphical nature allows for ready interpretation of all associations present. In this study, a small variable domain – ten pathologies studied in 14 variables – is modelled with a substantial amount of data, providing the ideal scenario for structure discovery multivariate analyses [25].

## Conclusions

The application of novel multi-dimensional machine learning methodology provided new insights into how typical pig pathologies are interrelated at batch level, assisting in elucidating theories on their biological associations. The results from this study could be also used to optimise abattoir inspection utilising risk based surveillance strategies. The methodology presented is a powerful hypothesis-generating exploratory tool, applicable to wide range of studies in veterinary research.

## Authors' contributions

MJSV conceived the study, carried out the statistical analyses and drafted the manuscript. FIL contributed to the conception of the study, wrote the statistical software required and assisted in drafting the manuscript. MN participated in its design and helped to draft the manuscript. SAE participated in its design and coordination and helped to draft the manuscript. GG helped to review the manuscript. All authors read and approved the final manuscript.

## Supplementary Material

Additional file 1 Data derived batch categorization for enzootic pneumonia and pleurisy. Click here for file
