# Supplementary material for: Identifying associations between pig pathologies using a multi-dimensional machine learning methodology
Source: BMC Vet Res. 2012 Aug 31;8:151. doi: 10.1186/1746-6148-8-151 (PMC3483212; doi:10.1186/1746-6148-8-151)
Supplement: Additional file 1 — Data derived batch categorization for enzootic pneumonia and pleurisy. [file 1746-6148-8-151-S1.doc]

# Supplementary material

### Data derived batch categorization for enzootic pneumonia and pleurisy.


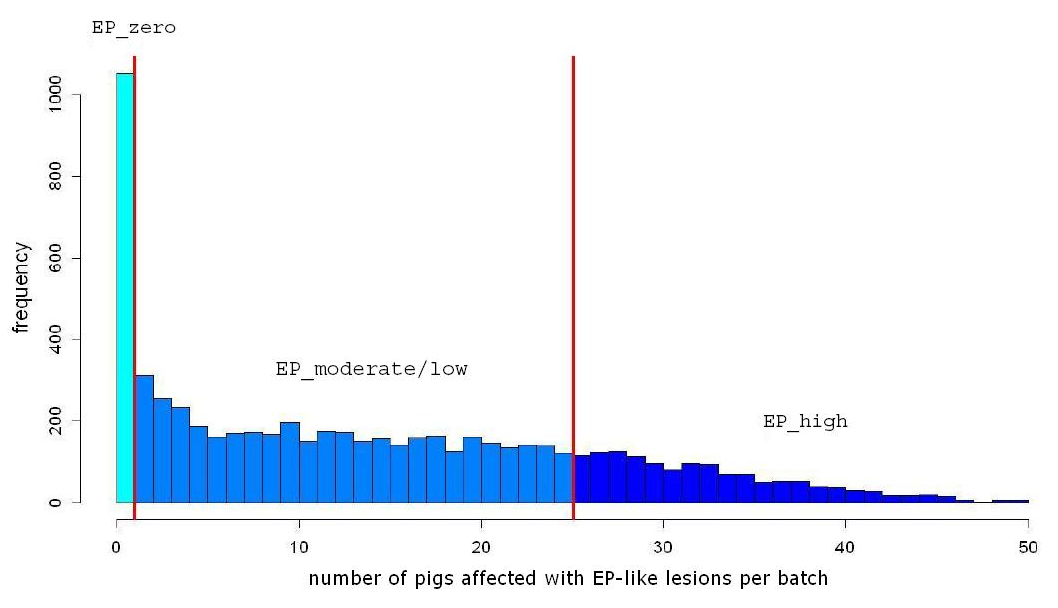


**Figure 1 (Supplementary material)**: Histogram of the number of pigs affected with EP-like lesions per batch (all batches consisted of 50 pigs inspected). The data are grouped in three categories. A distinctive section of the batches was reported with zero prevalence (EP zero). For the positive batches two additional groups were created, those with less/equal half (25) of the pigs affected (moderate/low prevalence) and those with more than half of the pigs affected (high prevalence).


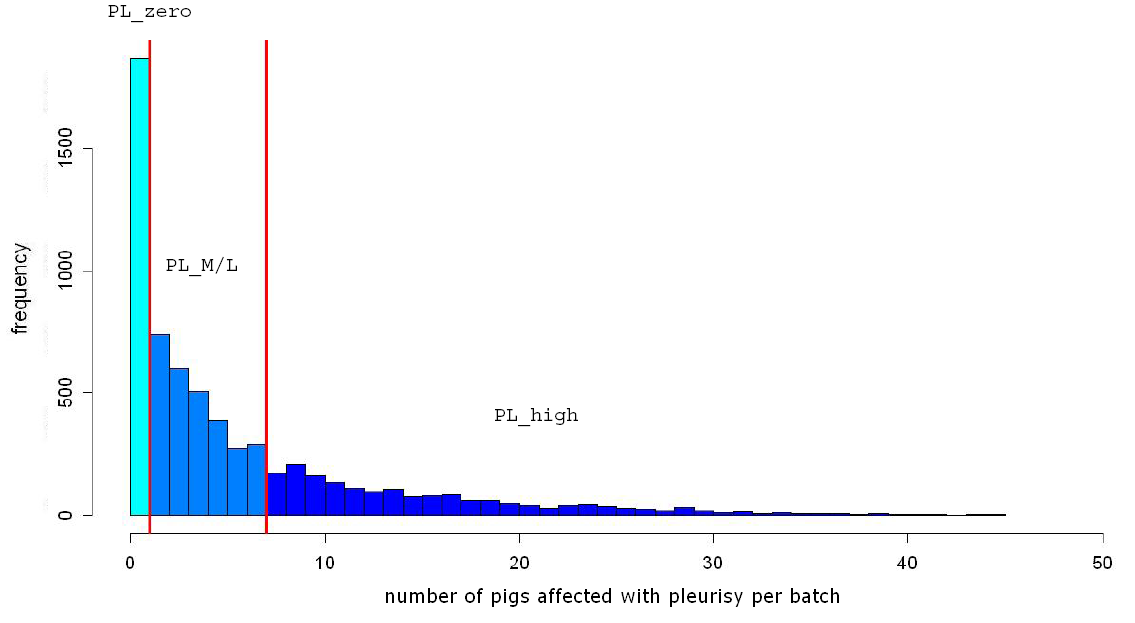


**Figure 2 (Supplementary material)**: Histogram of the number of pigs affected with pleurisy per batch (all batches consisted of 50 pigs inspected). The data are grouped in three categories. A distinctive section of the batches was reported with zero prevalence (PL Zero). For the positive batches a drop identified in the histogram when more than seven pigs were affected with pleurisy was used as a data derived cut-off point to group the batches, thus: less or equal to seven pigs affected (moderate/low prevalence) and more than seven pigs affected (high prevalence).
